# Comprehensive Analysis of Phagocytosis-Related Regulators to Aid Prognostic Prediction and Immunotherapy in Patients with Low-Grade Glioma

**DOI:** 10.1155/2022/4142684

**Published:** 2022-04-12

**Authors:** Jianwen Li, Qianrong Huang, Ligen Mo

**Affiliations:** ^1^College of Oncology, Guangxi Medical University, Nanning, Guangxi, China; ^2^Department of Neurosurgery, Guangxi Medical University Cancer Hospital, Nanning, Guangxi, China

## Abstract

Antibody-dependent cellular phagocytosis- (ADCP-) related regulators (PRs) have been confirmed an important role in immunotherapy. However, the characterization of specific PRs in low-grade glioma (LGG) has not been comprehensively explored. In this study, we retrieved RNA-seq and CRISPR-Cas9 data to identify specific PRs in LGG patients and constructed a PRs-signature using the LASSO-Cox algorithm. The ROC analysis and Kaplan-Meier analysis showed that PRs-signature had a good predictive effect, and the multivariate Cox regression analysis showed that PRs-risk scores were independent prognostic factors correlated with overall survival (OS). In addition, CIBERSORT, ssGSEA, and MCP counter algorithms were used to explore immune cell content in different risk groups, especially in the correlation between macrophages and specific PRs. Finally, mRNA expression was upregulated in the high-risk group compared with the low-risk group at most immune checkpoints and proinflammatory factors. In conclusion, we constructed a prediction model for prognostic management and revealed the cross-talk between specific PRs and immunotherapy in LGG patients.

## 1. Introduction

The most frequent primary central nervous system tumors are low-grade glioma, which arises from glial cells. Surgical excision, radiation, and chemotherapy are possibilities for glioma treatment. However, the overall survival (OS) remains low [1]. As a result, the major objective of therapy is to improve the overall survival (OS). It is vital to be able to identify high-risk patients and personalize treatment to them to attain this aim.

Phagocytosis is involved in several disease processes, including the clearance of apoptotic cells, cell regeneration, tumor monitoring, and removal of cellular debris following damage [2]. Meanwhile, autoimmunity and developmental abnormalities can occur when phagocytosis is out of balance [3]. In addition, to engulf various types of particles, phagocytes use diverse surface receptors and signaling cascades [4]. It is worth mentioning that monoclonal antibody therapies targeting tumor antigens drive cancer cell elimination in large part by triggering macrophage phagocytosis of cancer cells [5]. Therefore, the identification of antibody-dependent cellular phagocytosis- (ADCP-) related regulators has become important in tumor immunotherapy. Luckily, the development of the CRISPR-Cas9 system has enabled dramatically improved genome-scale knockout screens with high precision in mammalian cells [6]. Therefore, the researchers have performed a large-scale identification by this method for ADCP-related regulators (PRs). However, the prognostic correlation between PRs and LGG has not been thoroughly studied.

Therefore, our study was aimed at developing a novel prognostic signature based on the above PRs to predict OS in LGG patients. In addition, we further validated the tumor immune microenvironment and response to immunotherapy. In particular, the association of specific PRs with macrophages in LGG tissues was explored and whether PRs could be used to assess ADCP status.

## 2. Materials and Methods

### 2.1. Datasets and Data Preprocessing

A total of 1081 WHO grade II and III glioma samples (TCGA database [7]) and 103 normal cortical samples were included in the study (GTEx project [8]). The TCGA-LGG dataset (*n* = 506) was defined as the training cohort and the CGGA dataset (*n* = 575) as the validation cohort. It is worth noting that samples have been excluded with clinical information with non-LGG and incomplete follow-up information. Meanwhile, IMvigor210 [9], a cohort of atezolizumab (anti-PD-L1 antibody) for uroepithelial carcinoma, was extracted to evaluate the predictive value of our signature for immunotherapy. In addition, regulators of cancer cell phagocytosis were derived from 730 genes identified using the CRISPR-Cas9 method.

### 2.2. Identification and Validation of Signature Based on PR Expression

A list of differentially expressed PRs (*p* < 0.05, ∣log FC | >1) was identified as specific PRs in LGG based on RNA-seq data obtained from TCGA and GTEx database [10]. In parallel, we calculated the macrophage content in LGG tissues using the ssGSEA algorithm; subsequently, the Spearman analysis was used to further explore the correlation between specific PRs and macrophages. Among the specific PRs, they were screened by univariate Cox regression analysis with a threshold of*p* < 0.001and further screened by Kaplan-Meier survival curves and log-rank test. Subsequently, hub PRs were identified by LASSO regression analysis [11] and the multivariate Cox regression analysis. The risk score for each patient was calculated by multiplying the expression values of certain genes by their weights in the multivariate Cox model and then adding them together; the formula was as follows: ∑_*i*=1_^*n*^Coef_i_∗*x*_*i*_. The prognostic value of the PRs-signature was assessed by the TCGA and CCGA cohorts. PRs-signature for predicting survival was assessed by area under the curve (AUC) and receiver operating characteristic (ROC) curve. We calculated the risk score of each patient for determining the median value, which is used to select “high-risk” and “low-risk” groups. The Kaplan-Meier analysis was performed to compare differences in overall survival (OS) between different groups [12].

### 2.3. Comprehensive Analysis of Molecular and Immune Characteristics

KEGG and GO enrichment analyses were used to explore the possible biological processes of specific PRs [13]. To identify the immune characteristics of the TC samples, expression data were imported into CIBERSORT package to estimate the proportion of 22 immune cells [14], and the ssGSEA algorithm and MCP counter [15] algorithms were used for further validation. Finally, the mRNA expression levels of immune checkpoint and proinflammatory factors were analyzed in different groups.

### 2.4. Vitro Assays

Two grade III glioma cell lines (HS683 and SW1088) were purchased from American Type Tissue Culture Collection. siRNA targeting SDHC was purchased from Genepharm (Nanjing, China). Human glioma cell line U251 and human glial cell HEB were obtained from ATCC, cultured in MEM-EBSS and DMEM-EBSS basal culture medium (Biological Industries), and supplemented with 10% FBS (Biological Industries). The cells were cultured in a humid condition with 5% CO_2_ at 37°C. Isolation of total RNA from the cell lines was performed according to the instructions for the TRIzol reagent, and the purity and concentration of RNA were determined by MV3000 Microspectrophotometer (260/280 ratio). Equal amounts of RNA were then reversely transcribed to make complementary DNA. We used GAPDH as an internal control gene; reactions were carried out using the SYBR Green Premix Pro Taq HS qPCR Kit (Accurate Biology, AG11701). Finally, a BIO-RAD CFX96 Real-Time PCR Detection System was used for the determination of the target gene expression levels. Each sample was repeated three times. The relative abundance of each gene mRNA was calculated by the 2^−*ΔΔ*Ct^ method. Detailed experiment was carried out according to the methods in the previous reference [16]: siRNA-1 5′-GATTAAGAATCAAGTTCACTC-3′and siRNA-2 5′-GATCAACTTCTATGAACAGTA-3′and SDHC forward: 5′-GATCTACTACACGCCACGAAG-3′and reverse: 5′-GCGTTGCCACAGATACATC-3′.

### 2.5. Cell Proliferation Assay

The cell growth rate was detected by CCK-8 cell proliferation assays. The cells were seeded in a 96-well plate at the density of 1.0 × 104 cells per well. The cell viability was detected at four selected time points (0, 12, 24, and 48 hours). CCK-8 solution (10 *μ*L) was added to each well at indicated times and incubated for another 3 hours. The absorbance of each well was obtained from the PerkinElmer 2030 Victor X multilabel plate reader (PerkinElmer) at 450 nm.

### 2.6. Western Blot

All the proteins were extracted. The cell lysate was run on SDS-PAGE and then transferred to a PVDF membrane (MILLIPORE). The membrane was then blocked with 5% fat-free milk for 2 hours at room temperature. Next, the membrane was probed with a primary antibody at 4°C overnight. The primary antibodies against were SDHC (Abcam, Ab155999, 1 : 10000) and GAPDH (Abcam, Ab8245, 1 : 1000). After washing, the membrane was incubated with appropriate secondary antibodies (Bioswamp) at 4°C overnight as well. The membrane was stained with enhanced chemiluminescence reagent and visualized using the automatic chemiluminescence analyzer (Tanon).

### 2.7. Statistical Analysis

**Figure 1:**
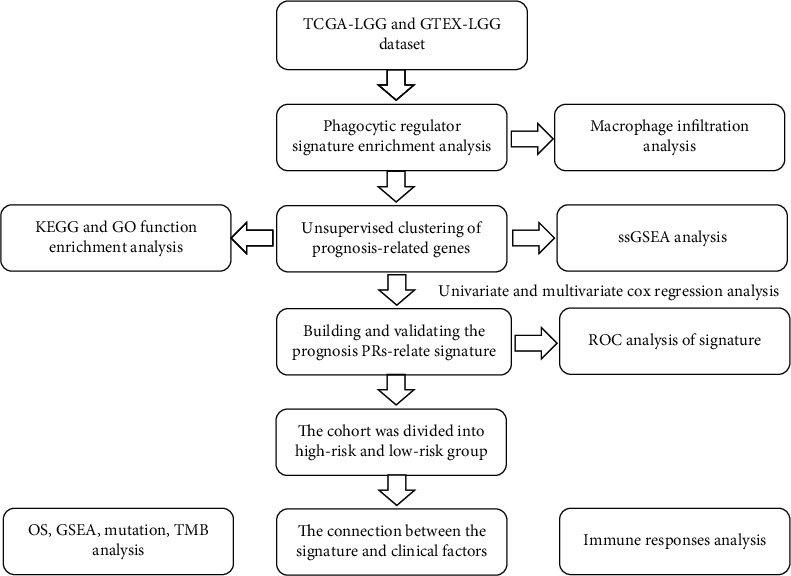
Workflow diagram. The specific workflow graph of data analysis.

All statistical analyses were performed using the R software (v.4.0.1). Detailed statistical methods for transcriptome data processing are covered in the above section. ∗∗∗, ∗∗, ∗, and ns refer to *p* < 0.001, <0.01, <0.05, and not significant, respectively. The overall flow diagram is shown in Figure 1.

## 3. Results

### 3.1. Specific PRs in LGG May Be Involved in the ADCP Process

**Figure 2:**
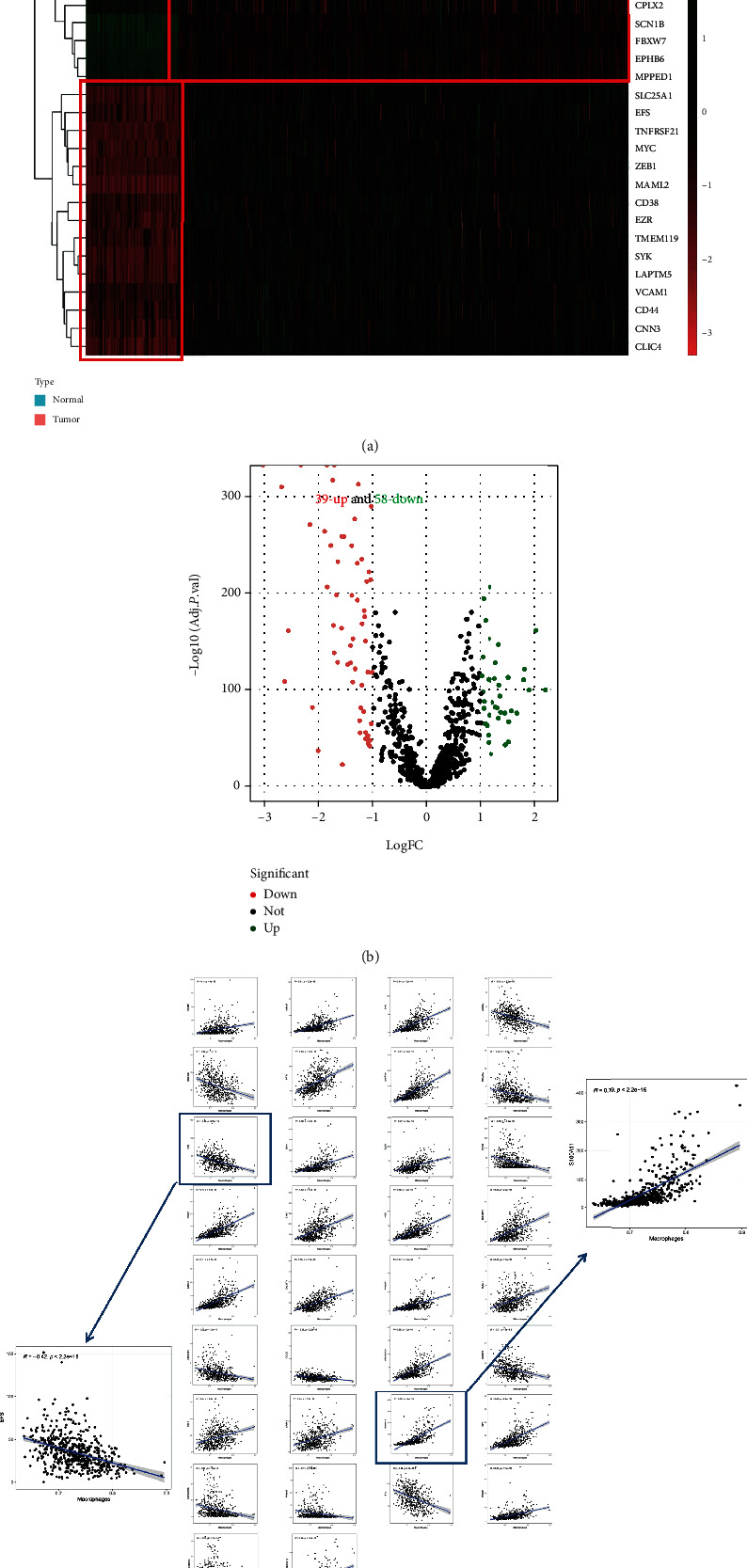
Identification of specific PRs in LGG. (a) The heatmap showed the top 10 specific PRs. (b) Specifically, the volcanic plot showed the distribution of upregulation and downregulation in specific PRs. (c) Scatter diagram of specific PRs correlated with macrophage content.

Monoclonal antibody therapies targeting tumor antigens trigger macrophages to engulf cancer cells [16], and regulators that block antigen-dependent cell phagocytosis (ADCP) have been identified using the CRISPR method. Therefore, we used differential gene screening analysis to identify specific PRs in LGG tissues, and we identified a total of 97 specific PRs in 506 LGG and 103 normal samples (Figure 2(a)). Among them, 39 upregulated genes and 58 downregulated genes were shown in the volcano plot (Figure 2(b)). Meanwhile, we used the ssGSEA algorithm to estimate the macrophage content in LGG tissues as a way to further explore the correlation between specific PRs and ADCP process. As shown in Figure 2(c), a total of 34 PRs were identified from 97 specific PRs that were strongly correlated with macrophage content (*p* < 0.001; ∣*r* | >0.3). Interestingly, EFS demonstrated the strongest negative correlation, while S100A11 showed the strongest positive correlation with macrophages in the LGG. Taken together, our data showed that specific PRs, especially in EFS and S100A11, are most likely to be involved in the ADCP process in LGG.

### 3.2. Identification of PRs Related to Biological Processes

**Figure 3:**
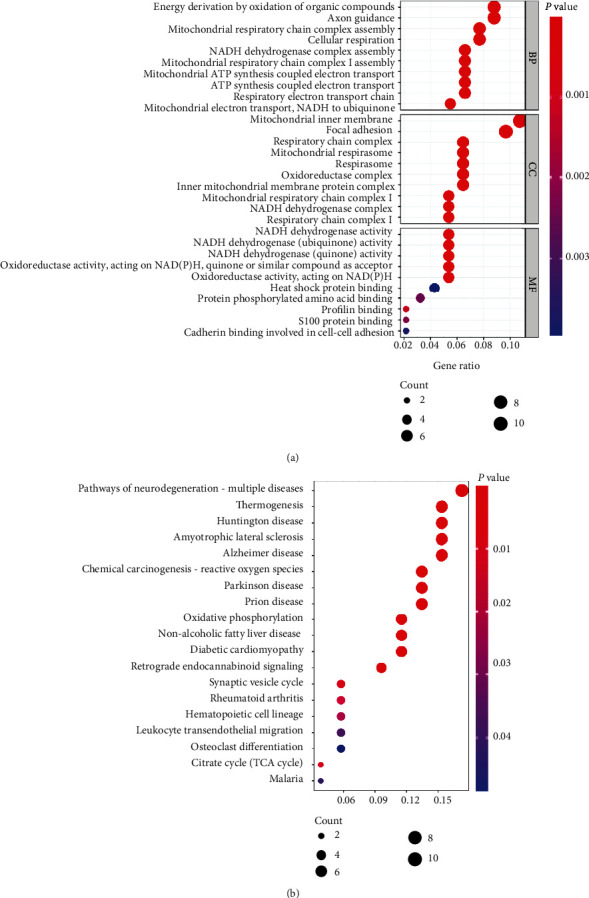
Enrichment analysis. (a) GO enrichment analysis about 97 specific PRs. (b) KEGG enrichment analysis about 97 specific PRs.

GO and KEGG enrichment analyses were performed to clarify the biological functions of specific PRs. GO enrichment analysis showed that 97 specific PRs were mainly related to mitochondrial respiratory chain complex assembly and NADH dehydrogenase complex assembly in BP section, respiratory chain complex and mitochondrial respiratory chain complex I in CC section, and NADH dehydrogenase (ubiquinone) activity in MF section (Figure 3(a)). In addition, KEGG enrichment analysis showed that related PRs were enriched in thermogenesis, oxidative phosphorylation, nonalcoholic fatty liver disease, etc. (Figure 3(b)).

### 3.3. Potential Prognostic Value of Specific PRs in LGG Patients

**Figure 4:**
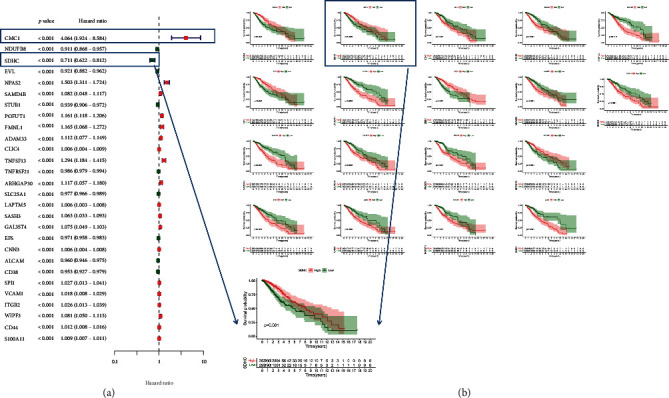
Potential prognostic value of each specific PRs. (a) The forest plot showed the prognostic specific PRs in the univariate Cox regression. (b) Kaplan-Meier analysis revealed 16 prognostic-specific PRs.

Firstly, a preliminary screening of 97 specific PRs using the univariate Cox regression analysis (*p* < 0.001) showed that 28 PRs were significantly associated with OS in LGG patients (Figure 4(a)). In detail, CMC1 (HR = 4.064) demonstrated a strong risk-indicating effect, and SDHC (HR = 0.711) showed a strong protective effect. In addition, we identified the above 28 PRGs again using the Kaplan-Meier survival analysis and log-rank test (*p* < 0.001), and finally 14 PRs proved to have strong prognostic value (Figure 4(b)). Notably, SDHC showed the best prognostic value within both screening methods.

### 3.4. Quantify the Predictive Value of PRs

**Figure 5:**
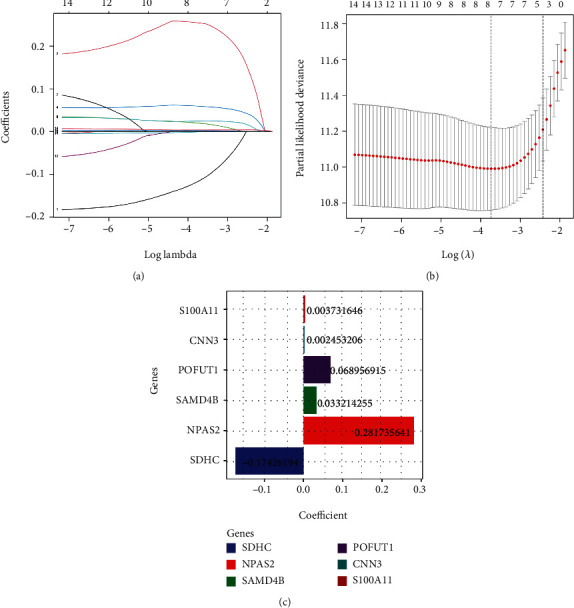
Quantify the predictive value of PRs on OS. (a and b) LASSO regression analysis. (c) The regression coefficients of each specific PRs participated in modeling.

The low phagocytic activity of macrophages and the expression of antiphagocytic factors in cancer cells are still obstacles to the targeted therapy for various cancers, and the associated dysregulation of PR expression can indirectly impact patient survival. Therefore, we attempted to quantify PRs for prognostic assessment of LGG patients in response to phagocytosis ability. Based on the 14 PRs mentioned above, we further removed redundant genes by LASSO regression (Figure 5(a)), and the prognostic model performed best when 8 PRs were used (Figure 5(b)). In addition, further stepwise multivariate Cox regression identified 6 PRs to be involved in the calculation of risk scores, and correlation coefficients were determined (Figure 5(c)). The formula was PRs − risk score = (0.0037 × expression level of S100A11) + (0.0024 × expression level of CNN3) + (0.0689 × expression level of POFUT1) + (0.0332 × expression level of SAMD4B) + (0.2817 × expression level of NPAS2) + (−0.1743 × expression level of SDHC).

### 3.5. Prognostic Value of PRs-Risk Score in LGG Patients

**Figure 6:**
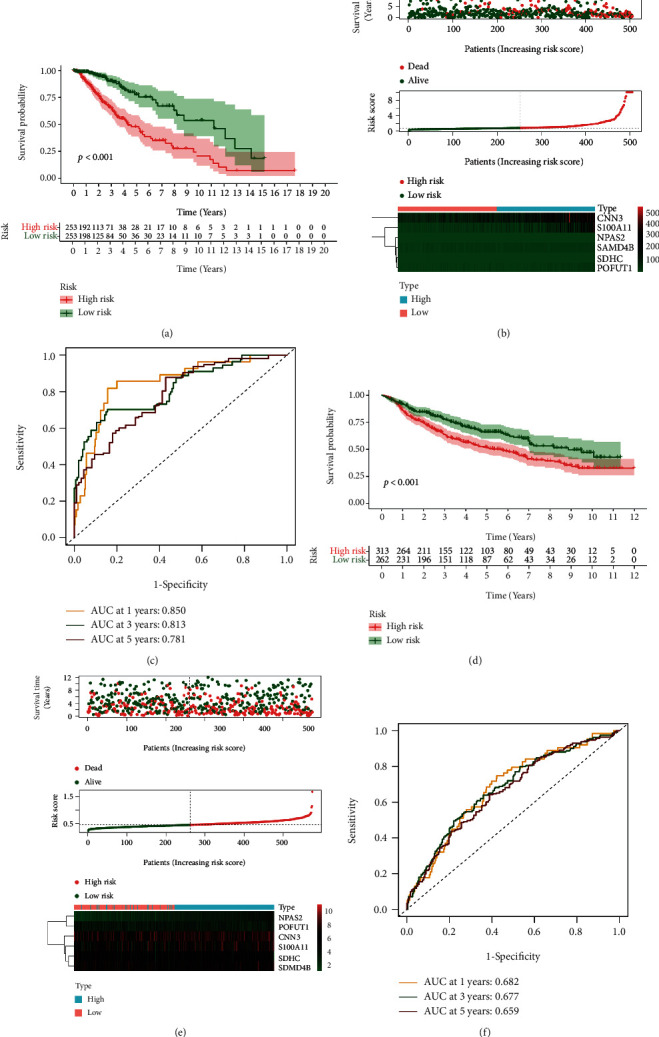
Validation of predictive value. (a) Kaplan-Meier analysis in the training cohort. (b) Survival distribution in the training cohort. (c) ROC analysis for 1, 3, and 5 years in the training cohort. (d) Kaplan-Meier analysis in the testing cohort. (e) Survival distribution in the testing cohort. (f) ROC analysis for 1, 3, and 5 years in the testing cohort.

**Figure 7:**
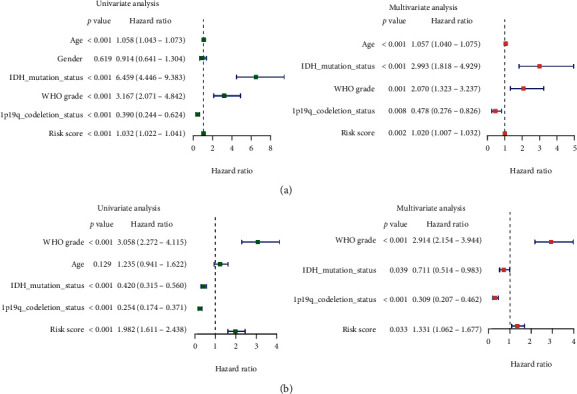
Independent prognostic value. (a) Univariate Cox regression in age, gender, IDH mutation status, WHO grade, 1p19q codeletion status, and PRs-risk score. (b) Multivariate Cox regression in the above clinical features.

All patients in the TCGA and CGGA cohorts were differentiated into low-risk and high-risk groups based on the median PRs-risk score in the TCGA-LGG cohort. The Kaplan-Meier survival analysis showed significant differences in OS between the low- and high-risk groups (*p* < 0.05, Figures 6(a) and 6(d)); specifically, the OS time was shorter in the high-risk group than in the low-risk group. In addition, the risk score distribution demonstrated that patients in the high-risk group had a greater likelihood of death (Figures 6(b) and 6(e)). ROC analysis of the TCGA cohort showed that the PRs-risk score was a good predictor (1-year AUC = 0.850, 3-year AUC = 0.813, and 5-year AUC = 0.781), as shown in Figure 6(c). Similarly, good predictive power was shown in the CGGA cohort (1-year AUC = 0.682, 3-year AUC = 0.677, and 5-year AUC = 0.659), as shown in Figure 6(f). Combined with the clinical information, we performed the Cox regression analyses to assess whether PRs-risk score could be used as an independent predictor for patients with LGG. As shown in Figure 7, the univariate Cox regression analysis in the different cohorts showed that PRs-risk score was significantly associated with OS in all LGG cases (*p* < 0.05). In addition, the multivariate Cox regression analysis showed that PRs-risk score was an independent risk factor associated with OS as well (*p* < 0.05).

### 3.6. Correlation of PRs-Risk Score and Clinical Subgroups

**Figure 8:**
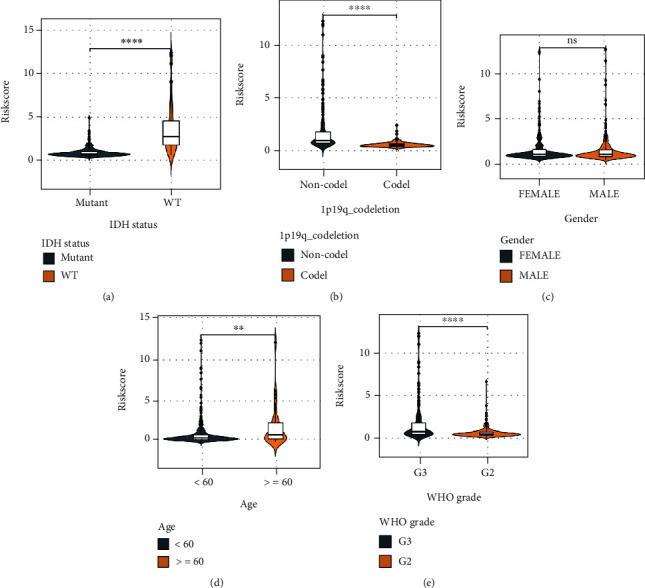
Clinical correlation analysis. Difference of PRs-risk score in IDH status (a), 1p/19q codeletion status (b), gender (c), age (d), and WHO grade (e). ^∗^*p* < 0.05, ^∗∗^*p* < 0.01, and ^∗∗∗^*p* < 0.001.

**Figure 9:**
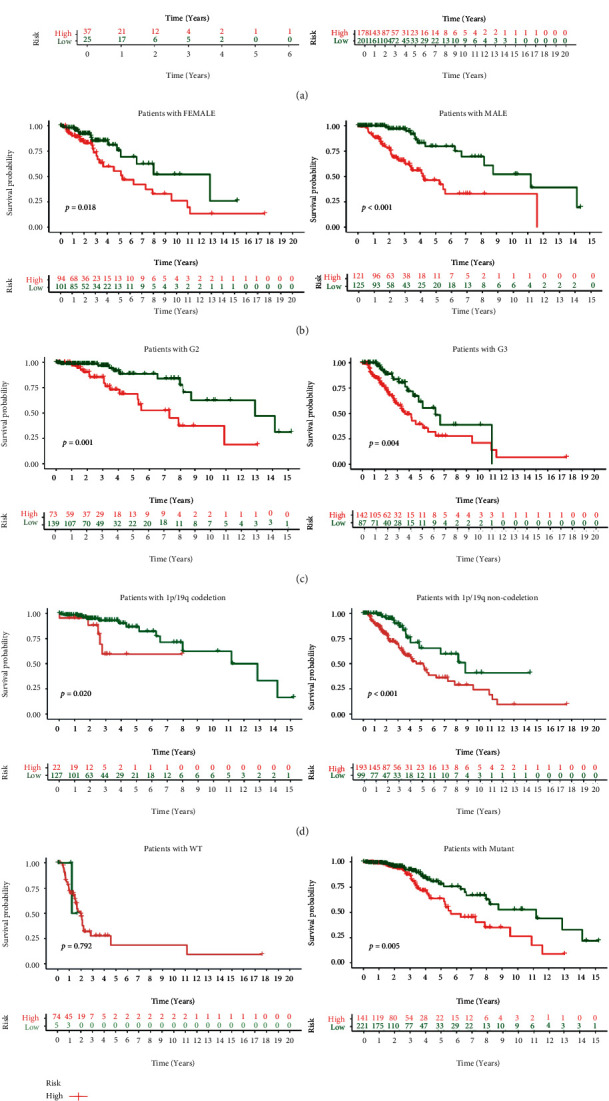
Kaplan-Meier analysis in different clinical subgroups. Kaplan-Meier analysis in age subgroups (a), gender subgroups (b), WHO grade subgroups (c), 1p/19q codeletion subgroups (d), and IDH status (e).

To explore the potential correlation between PRs-risk score and clinicopathological factors, we performed correlations between age, WHO grade, IDH status, and 1p/19q mutation status. The results showed that among the clinical subgroups, IDH-WT subgroup (Figure 8(a)),1p/19q non-codeletion subgroup (Figure 8(b)), elderly subgroup (Figure 8(d)), and G3 group (Figure 8(e)) had higher PRs-risk scores. In contrast, no statistically significant differences in risk scores were observed in gender subgroups (Figure 8(f)). In addition, we performed the Kaplan-Meier analysis for each subgroup, and PRs-risk scores showed a risky indication in the young group (Figure 9(a)), the male/female groups (Figure 9(b)), the G2/G3 groups (Figure 9(c)), the 1p/19q mutation/nonmutation groups (Figure 9(d)), and the IDH mutation group (Figure 9(e)), with higher-risk patients having a smaller likelihood of survival. Moreover, there was no significant survival difference in the older group as well as in the IDH-WT group, but it is worth noting.

### 3.7. Immune Cell Infiltration in Different PRs-Risk Groups

**Figure 10:**
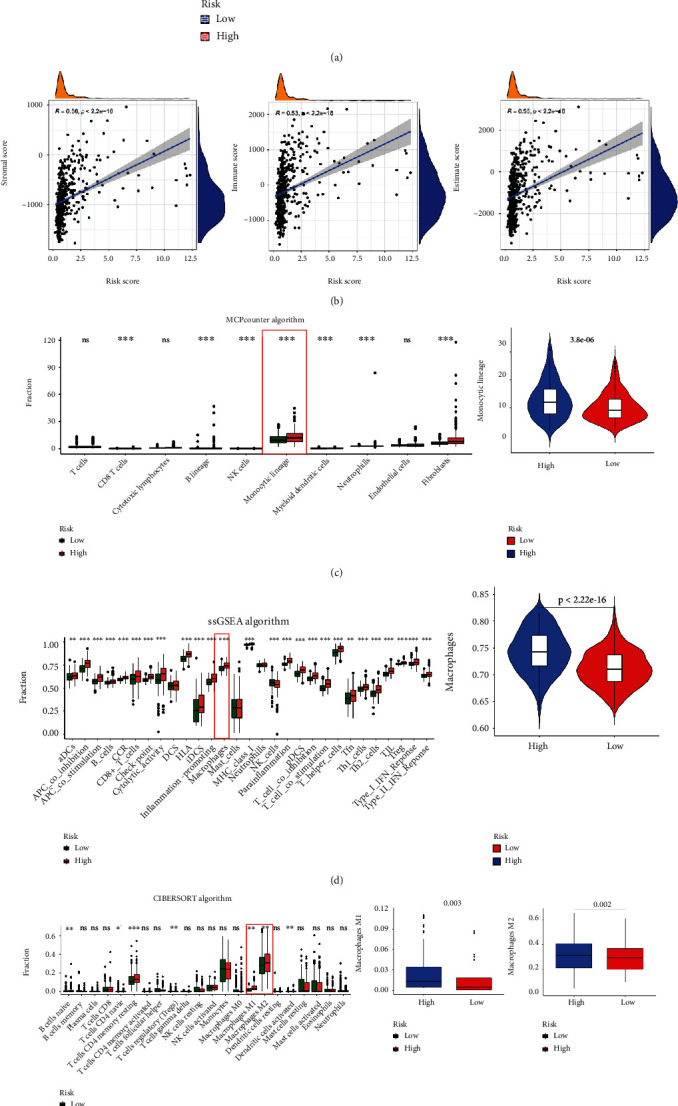
Estimate, MCP counter, ssGSEA, and CIBERSORT algorithms to explore immune status in different PRs-risk groups. (a) Difference in stromal score, immune score, and ESTIMATE score in different PRs-risk groups. (b) Correlation analysis between PRs-risk score and three scores. (c) The difference in immune cells is calculated by the MCP counter algorithm. (d) The difference in immune cells is calculated by the ssGSEA algorithm. (e) The difference in immune cells was calculated by the CIBERSORT algorithm. ^∗^*p* < 0.05, ^∗∗^*p* < 0.01, and ^∗∗∗^*p* < 0.001.

Firstly, we assessed tumor microenvironment (TME) in the LGG tissues using the ESTIMATE algorithm, with a higher stromal score, immune score, and estimate score in the high-risk group (Figure 10(a)). We then excluded three samples with significantly outlier risk scores (risk score > 100) and performed a Spearman correlation analysis between PRs-risk score and TME scores, which showed a strong positive correlation (*r* > 0.5, *p* < 0.05) between risk score and TME scores (Figure 10(b)). We used the MCP counter algorithm to profile the content of an immune cell in LGG tissue (Figure 10(c)). Interestingly, we found a more pronounced distribution of scores in the monocyte lineage and higher monocyte lineage scores in the high-risk subgroup compared to the low-risk group. In addition, we performed a detailed analysis of the monocyte lineage using the ssGSEA algorithm and found that within the monocyte lineage, macrophages were still more abundant in the high-risk group (Figure 10(d)). Finally, the CIBERSORT algorithm illustrated that macrophage M1 was higher in the lower-risk group compared to the high-risk group, while M2 was higher in the high-risk group (Figure 10(e)). Taken together, the results of the three algorithms provided another value that risk score may be indicative of the macrophage content and the status of the ADCP process induced by PRs.

### 3.8. Risk Score as an Indicator Aimed to Estimate the Situation of Immune Checkpoint and Proinflammatory Factors

**Figure 11:**
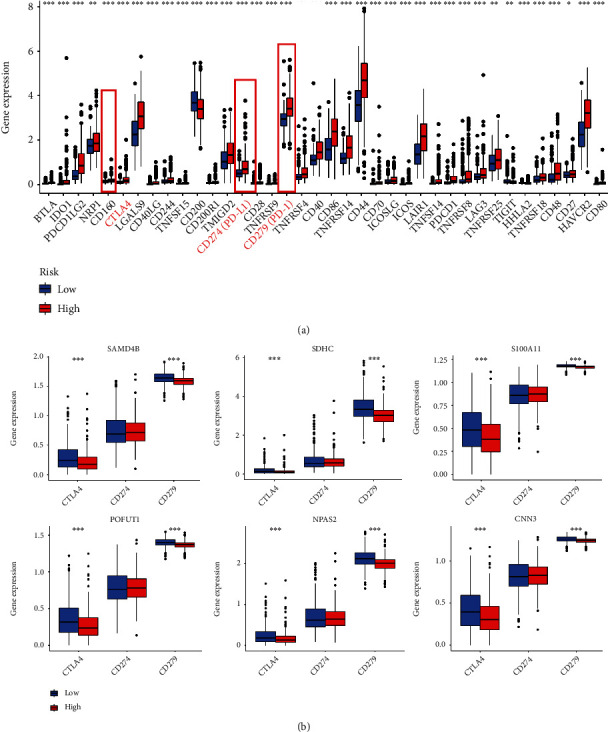
Exploring potential immune checkpoints in different PRs-risk groups. (a) Significant immune checkpoint expression in different risk groups. (b) The difference in CTLA4, CD279, and CD274 expressions in S100A11, CNN3, POFUT1, SAMD4B, NPAS2, and SDHC groups.

**Figure 12:**
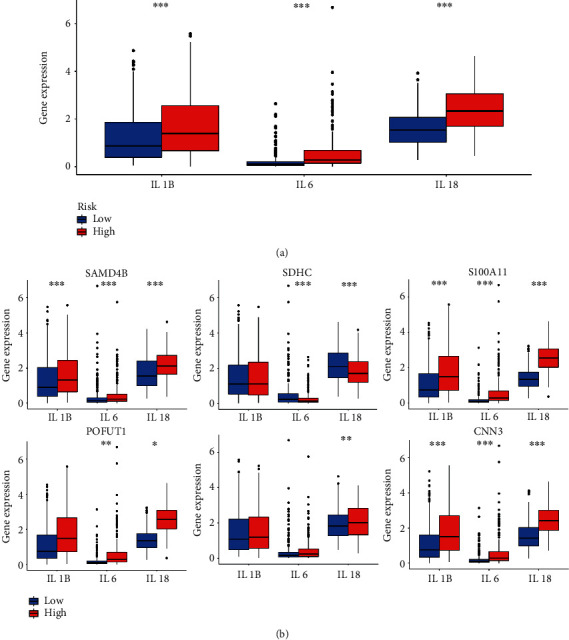
Exploring proinflammatory factors in different PRs-risk groups. (a) Significant proinflammatory factor expression in different risk groups. (b) The difference in IL1B, IL6, and IL18 expressions in S100A11, CNN3, POFUT1, SAMD4B, NPAS2, and SDHC groups.

To explore the relationship between immune checkpoints and risk score as well as the 6 selected PRs, immune checkpoint expression levels were calculated for high- and low-risk groups based on the TCGA-LGG database (Figure 11(a)). Interestingly, among the immune checkpoints, including PD-1, PD-L1, and CTLA4, which are currently commonly used in LGG clinics, mRNA expression was upregulated in the high-risk group compared to the low-risk. In addition, PD-1 and CTLA4 expression levels were significantly different between the high and low subgroups of the 6 selected PRs (Figure 11(b)). In detail, the high expressing PR group had lower levels of immune checkpoint expression. Numerous studies have shown that chronic inflammation has an important role in immune cell infiltration and major proinflammatory factors, including interleukin 1*α* (IL-1A), interleukin 1*β* (IL-1B), interleukin 6 (IL-6), and interleukin-18 (IL-18) [17]. Hence, we explored the association of three major proinflammatory factors with risk scores. The results showed that the expression levels of IL-1B, IL-6, and IL-18 were significantly higher in the high-risk group (*p* < 0.001, Figure 12(a)). Meanwhile, three proinflammatory factors showed high-expression levels in the SAMD4B, S100A11, and CNN3 high-expression groups; IL-6 and IL-18 were expressed at higher levels in the low SDHC expression group than in the high-expression group, and IL-6 and IL-18 were expressed at higher levels in the high POFUT1 expression group than in the low-expression group. In addition, the expression levels of IL-18 in the high NPAS2 expression group were lower than those in the low-expression group (Figure 12(b)).

### 3.9. Validating the Benefit of the Risk Score in ICI Treatment

**Figure 13:**
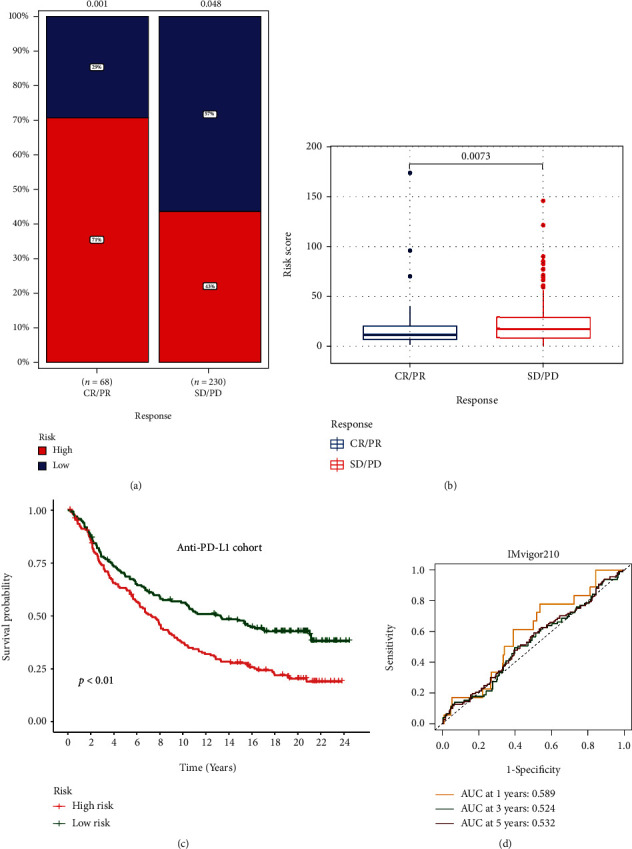
The assessment of immunotherapy. (a and b) Difference of risk score in different treatment response groups. (c) Kaplan-Meier survival analysis of anti-PD-L1 cohort (IMvigor210). (d) ROC analysis of different risk signatures in TCGA-TC cohort.

We assessed the prognostic value of PRs-signature in cohorts treated with anti-PD-L1. As shown in Figure 13(a), we found significant differences between the high- and low-risk score groupings in the CR/PR and SD/PD cohorts. In addition, patients in the SD/PD cohort had higher PRs-risk scores (Figure 13(b)). Meanwhile, patients with low PRs-risk scores had better OS than high PRs-risk scores (Figure 13(c)). Unfortunately, our PRs-signature may be a poor predictor of OS at 1, 3, and 5 years in the IMvigor cohort (Figure 13(d)), but the role of PRs in assessing response to ICI treatment should not be ignored.

### 3.10. Validation of Hub Prognostic PR In Vitro

**Figure 14:**
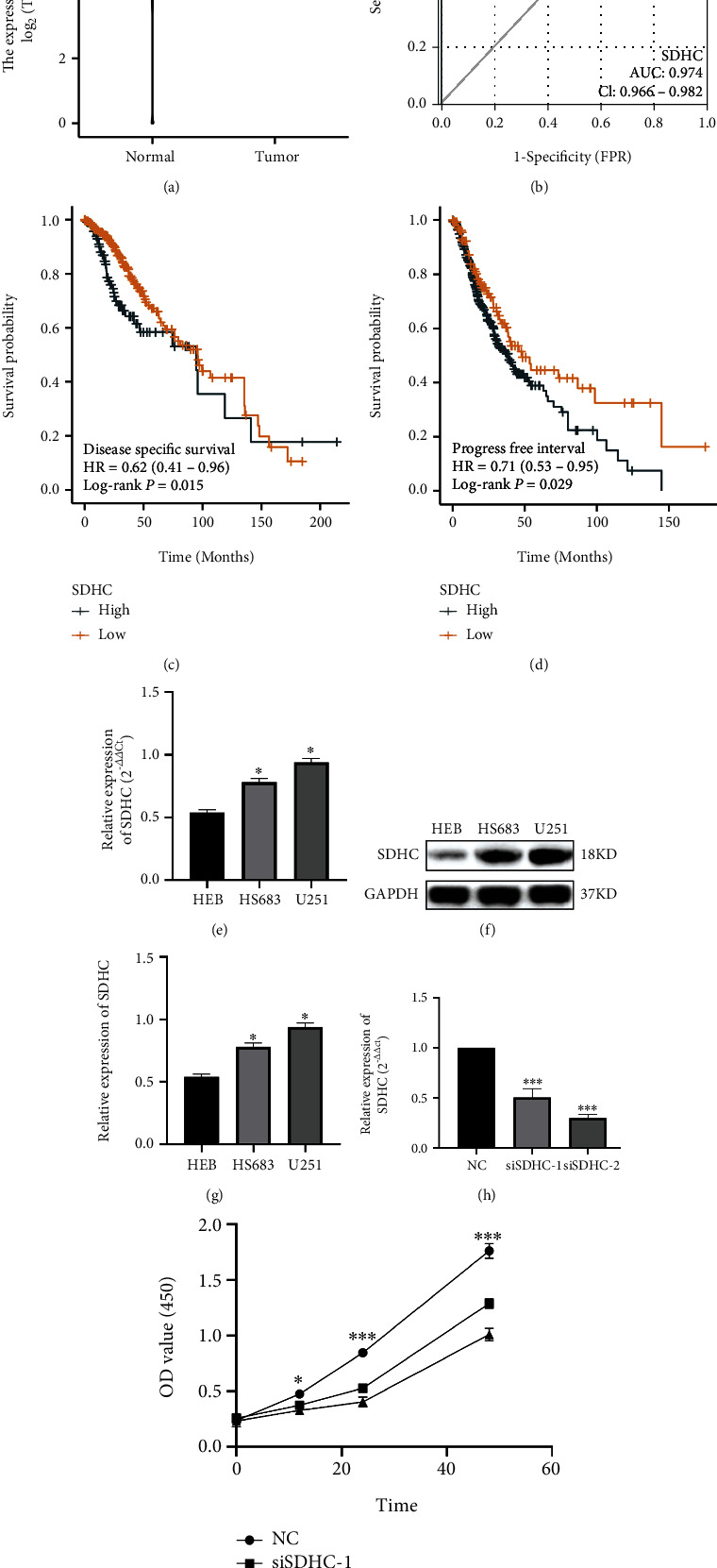
Validation of hub prognostic PR in vitro. (a) Differential expression of SDHC in normal and abnormal samples. (b) ROC analysis for diagnosis LGG. (c) Kaplan-Meier survival analysis for disease-specific survival. (d) Kaplan-Meier survival analysis for the progress-free interval. (e) SDHC mRNA expression in HEB, HS683, and U251 cell lines. ^∗∗∗^*p* < .001. (f and g) SDHC mRNA expression in HEB, HS683, and U251 cell lines. ^∗∗∗^*p* < .001. (h) RT-qPCR analysis of the cells with SDHC knockdown. (i) Knockdown of SDHC suppressed proliferation of U251 cells indicated by CCK-8 assay.

In the above, we found that SDHC showed the best prognostic value within both the Cox regression analysis and Kaplan-Meier analysis (Figure 2(c)). Hence, we further studied the expression, biological function, and diagnostic value of SDHC in LGG. SDHC is significantly upregulated in LGG tissues (Figure 14(a)) and has a good predictive value for the diagnosis (Figure 14(b)). In addition to being a predictor of overall survival, SDHC has excellent prognostic value for disease-specific survival (Figure 14(c)) and progress-free interval (Figure 14(d)). We also found that SDHC was significantly elevated in glioma cell lines compared to normal glial cells, so we knocked down the expression of SDHC in the U251 cell line and found that the proliferation rate of cells in the knockdown group was significantly lower compared to the untreated group, suggesting that SDHC is a poor prognostic factor that may be associated with affecting glioma proliferation (Figures 14(e)–14(i)).

## 4. Discussion

Phagocytosis is a multistep cellular process that includes target cell identification, cytophagy, and lysosomal digestion, all of which are influenced by target cell and receptor-ligand interactions [6]. Although healthy normal tissues and cells have inherited the ability to avoid self-elimination by phagocytes through the expression of antiphagocytosis molecules, cancer cells depend even more on similar mechanisms to evade immune eradication [18]. It is becoming increasingly clear that tumor cell phagocytosis and subsequent immune recognition are controlled by multiple inhibitory and stimulatory signals that must be considered to generate an optimal antitumor response. Innovative approaches using genetic screening strategies to identify key regulators of phagocytosis under physiological conditions and nontumorigenic pathologies can be extended to the oncology setting. For example, recent studies using genome-wide CRISPR screens have identified previously unknown regulators of phagocytosis that are important in amyloid *β* clearance in Alzheimer's disease. How these phagocytic regulators work in concert to regulate the clearance of tumor cells by professional phagocytes at different stages of tumorigenesis and in different types of cancer remains to be elucidated. From a clinical perspective, how to incorporate phagocytic checkpoint blockers/stimulators into the current cancer immunotherapy paradigm needs further evaluation. At the outset, targeting phagocytic checkpoints should complement existing T-cell immune checkpoint inhibitors to maximize antitumor responses. For example, tumors with low PD-L1 levels, which are less sensitive to blockade of the PD-1/PD-L1 axis, may be more sensitive to CD47-SIRP*α* interference. Similarly, adaptive immunotherapy relies on the generation of specific T-cell clones that recognize tumor-associated neoantigens, which correlates with the degree of tumor cell genomic alteration, whereas phagocytic checkpoint blockade appears to be effective also in cancers with the low mutational burden (e.g., AML). Therefore, comprehensive analysis of PRs is essential for immunotherapy and prognosis prediction of LGG. In this study, we constructed a robust PRs-score to predict the prognosis of LGG patients using the PRs-signature. For evaluation of treatment effectiveness, our PRs-signature can not only estimate the effect of immunotherapy but also preliminarily explore the potential association between specific PRs and ADCP status. Especially, in exploring the immune microenvironment, we found an important correlation between PRs-risk scores and M1/M2 type macrophages. Finally, we knocked down the hub prognostic PR (SDHC) and found that proliferation was significantly inhibited. Immunotherapies represented by the PD-1/PD-L1 and CTLA-4/B7 pathways have shown significant clinical efficacy in a variety of cancer types. However, only a small proportion of patients show a good response to anti-PD1-PD-L1 or anti-CTLA-4/B7 treatment. The overexpression of PD-L1 and CTLA-4 is an important suppressor of antitumor immunity and is associated with better therapeutic response and increased clinical benefit. Given the unprecedented predictive value of PD-L1 and CTLA-4 expression in immunotherapy, investigating the different regulators of PD-L1 and CTLA-4 expression that may influence immunotherapeutic efficacy will contribute to the individualized clinical management of cancer patients [19, 20]. This study identified 6 PRs with prognostic value in risk signature, including S100A11, CNN3, POFUT1, SAMD4B, NPAS2, and SDHC. S100A11 is a calcium-binding protein that belongs to the S100 family [2]. The upregulation of S100A11 has been found to accelerate tumor cell proliferation, migration, and invasion by activating several signaling pathways [21, 22]. Interestingly, S100A11 has also been discovered as a tumor-derived EVP protein implicated in eliciting immunological responses, suggesting that it is important in tumor immunity [23]. In addition, the protein encoded by CNN3 regulates cytoskeletal organization and actin interactions through efficient binding to F-actin [24]. A bioinformatics study has now confirmed CNN3 as a promising immunotherapeutic target in glioma [25]. In breast [26] and colorectal malignancies [27], NPAS2 has also been discovered as a unique predictive biomarker. Furthermore, in colorectal cancer, suppressing NPAS2 expression boosts cell proliferation and invasion, indicating that NPAS2 plays an important tumor-suppressive role. Although most of the above-mentioned genes are involved in the progression of cancer, how to affect the development of LGG and ADCP has not been well explained. Thus, this study lays the foundation for our future in vivo and in vitro experiments. Thus, phagocytic checkpoints may provide an alternative strategy for treating unresponsive or refractory tumors to conventional cancer immunotherapy or given concurrently with adaptive immune checkpoint inhibitors to improve overall patient response rates. Finally, it is also important to strike a balance between efficacy and toxicity. Unlike adaptive immune responses, which are limited to some extent by self-tolerance, innate responses are less specific and therefore more susceptible to damage by normal tissues. This is particularly important because phagocytic checkpoint inhibitors may be used in combination with other immunomodulatory agents, such as TLR or STING agonists, cytokines, or systemic chemotherapy. However, targeting phagocytic checkpoints provides new avenues for gaining insight into tumor-mediated immune evasion mechanisms and developing more effective therapies that can bridge the innate and adaptive immune systems for the benefit of cancer patients.

However, this study only used the data from the public database TCGA and CGGA to construct the model, and there was no condition to collect our data to validate PRs-signature, which was a limitation of our study. In addition, we only have a focus on SDHC in LGG cell lines, and experimental validation needs to be conducted in the future.

## 5. Conclusion

We constructed an accurate and robust PRs-signature for predicting the prognosis of patients with LGG. On this basis, we revealed the cross-talk between specific PRs and immunotherapy in LGG patients. In particular, different risk groups may represent different ADCP statuses in LGG patients. Comprehensively, these will shed light on the development of novel therapeutic strategies and render survival benefits for LGG patients.

## Data Availability

The following information was supplied regarding data availability: data is available at the TCGA (https://portal.gdc.cancer.gov/) and CGGA (http://www.cgga.org.cn/).
